# A panoramic view of technological landscape for bioethanol production from various generations of feedstocks

**DOI:** 10.1080/21655979.2022.2095702

**Published:** 2023-07-04

**Authors:** Arti Devi, Somvir Bajar, Priyanka Sihag, Zaheer Ud Din Sheikh, Anita Singh, Japleen Kaur, Narsi R. Bishnoi, Deepak Pant

**Affiliations:** a Department of Environmental Sciences, Central University of Jammu, Jammu and Kashmir, India; b Department of Environmental Sciences, J.C. Bose University of Science and Technology, YMCA, Faridabad, Haryana, India; c Department of Environmental Science & Engineering, Guru Jambheshwar University of Science and Technology, Hisar, Haryana, India; d Separation and Conversion Technology, Flemish Institute for Technological Research (VITO), Mol, Belgium; e Department of Environmental Studies, Central University of Haryana, Jant-Pali, Mahendergarh, Haryana, India

**Keywords:** Feedstock, generations, biomass, conversion, bioethanol

## Abstract

Bioethanol is an appropriate alternate energy option due to its renewable, nontoxic, environmentally friendly, and carbon-neutral nature. Depending upon various feedstocks, bioethanol is classified in different various generations. First-generation ethanol created a food vs fuel problem, which was overcome by second-generation, third-generation and fourth-generation ethanol. The considerable availability of lignocellulosic biomass makes it a suitable feedstock, however, its recalcitrant nature is the main hurdle in converting it to bioethanol. The present study gives a comprehensive assessment of global biofuel policies and the current status of ethanol production. Feedstocks for first-generation (sugar and starch-based), second-generation (lignocellulosic biomass and energy crops), third-generation (algal-based) and fourth-generation (genetically modified algal biomass or crops) are discussed in detail. The study also assessed the process for ethanol production from various feedstocks, besides giving a holestic background knowledge on the bioconversion process, factors affecting bioethanol production, and various microorganisms involved in the fermentation process. Biotechnological tools also play a pivotal role in enhancing process efficiency and product yield. In adddition, most significant development in the field of genetic engineering and adaptive evolution are also highlighted.

## Highlights

- Global biofuels policies on bioethanol production were presented
- Technological integrations of 1G, 2G, 3G and 4G bioethanol were discussed
- Various factors affecting bioethanol production were mentioned
- Techno-economic analysis and LCA of bioethanol production are discussed

## GRAPHICAL ABSTRACT

**Figure:**
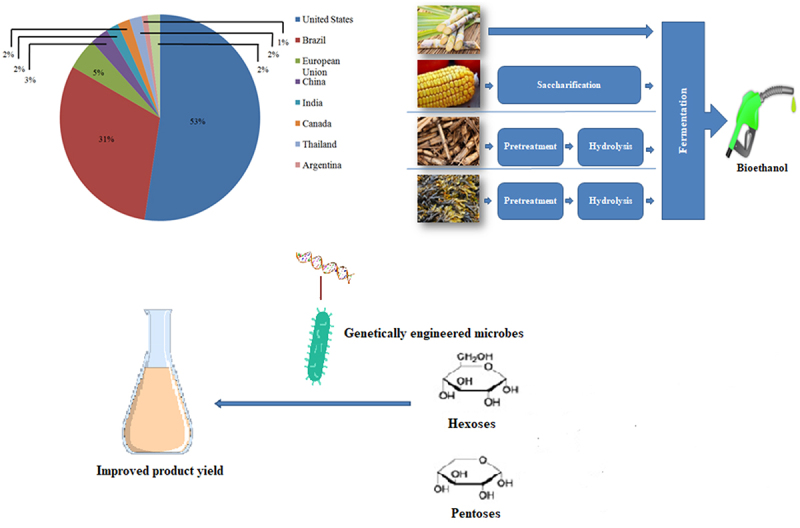

## 1. Introduction

Biofuels are gaining global attention due to energy security and environmental concern raised by fossil fuel burning [1] [2]. Fossil fuels are the fuel of choice, but their burning causes emission of greenhouse gas (GHG) emissions, climate change, global warming, biodiversity loss, and rising sea levels [3–6]. Moreover, it is estimated that by 2040, global energy use will elevate by more than 28% [7]. Renewable energy is an effective alternative that can fulfil the demand of the increasing human population and climate change and reduce the dependency on fossil fuels [8]. At the twenty-sixth Conference of Parties (COP26), countries made ambitious commitments to reaching net-zero emissions by around mid-century, on phasing out coal power and to end subsidizing unabated fossil fuels by the end of 2022 [9]. Biofuels are contemplated as a fascinating substitute to fossil fuels from the economic, environmental, and social points of view [10]. Many developed countries attempted to implement biofuel usage over fossil fuels and increase their dependency on bioethanol. Bioethanol as biofuel has an extensive history in the transportation field afetr being employed in internal combustion engines by Germany and France in 1984. Even before, Brazil started bioethanol as fuel utilization in the 1930s while the United States and Europe in the early 1900, however, the main focus started in the 1980s due to the oil crisis problem.

Life Cycle Analysis (LCA) on biofuel stated that carbon dioxide emission from plant growth, harvesting, and processing for biofuel production and its utilization in fuel engines is much lower than petrochemical fuels [11]. As per Renewable Fuel Association 2021, ethanol use in gasoline in 2020 decreases the emission of Greenhouse Gases (GHG) by 47.3 million metric tons from the transportation sector, which is equivalent to removal of emission from 12 coal fired power plant annually or phaseout of 10.1 million cars from roads for a year. The global bioethanol production in 2020 has been severely impacted due to the COVID-19 crisis and reached probably 98 billion liters, which was lesser than the previous year’s production, i.e. 115 billion liters. A fall of 15% was observed mainly due to the decreased output from the United States and Brazil [12]. The United States (US) and Brazil are still the global leader in term of ethanol production and, sharing about 82% of the whole bioethanol production in 2021 [13]. Each country has its biofuel policies, which are reviewed from time to time. Up to a particular percentage, bioethanol can be employed in unmodified petrol engines. Toxicity and emissions from ethanol are much lesser than fuels like diesel and petroleum. Ethanol can be manufactured from various feedstocks like starch, sugar, and cellulosic material [14]. Bioethanol is produced from distinct substrates worldwide, depending upon the availability and maturity of production technology. Depending on the feedstock, bioethanol production is divided into four different generations. The 1^st^ generation (1G) consists of edible crops that gave rise to conflict between food and fuel. While, the 2^nd^ generation (2G) was promoted with use non-food substrates comprising the residual part of edible crops or lignocellulosic biomass or energy crops. The third generation (3G) used the algal biomass, while the fourth generation (4G) utilized an engineered photosynthetic crop that captured carbon dioxide for growth and converted it into fuel. The search for the most sustainable and economical technology for converting bioresources has continued to get more production and yield of ethanol. The present review entails the current status of ethanol production worldwide, global biofuel policies, and different technologies used in ethanol production from various feedstocks, along with the microorganisms involved in the production process. The current review also assessed the factors (physical and nutritional) affecting ethanol production and advancements in biotechnological tools like genetic engineering and adaptive evolution in this field.

## 2. Present scenario of global bioethanol production

According to the International Energy Agency (IEA), a 3% increase in ethanol production was noticed globally since February 2020, with about 104 billion liters of its production. This production enhancement was mainly visualised by the US, European Union (EU), and China. With an average growth rate of 2% per annum, it will reach to about 119 billion liters by 2023 [15]. Daily ethanol production in 2021 was around 152.6 million liters, which was more than the 144.6 million liters in 2020, as predicted by IEA. Furthermore, it is expected to reach 157.4 million liters/day in 2022. It was also estimated that the daily ethanol blending wills reach to around 143.1 million liters in 2021 and 146.3 million liters in 2022. Both the predictions were escalated and in an another estimate in May 2021, Short Term Energy Outlook (STEO) forecasted that daily ethanol blending in 2021 will be 141.5 million liters and will again reach to 144.7 million liters in next year; as compared to its consumption in blending of 130.4 million liters per day in 2020 [16].

**Figure 1.**
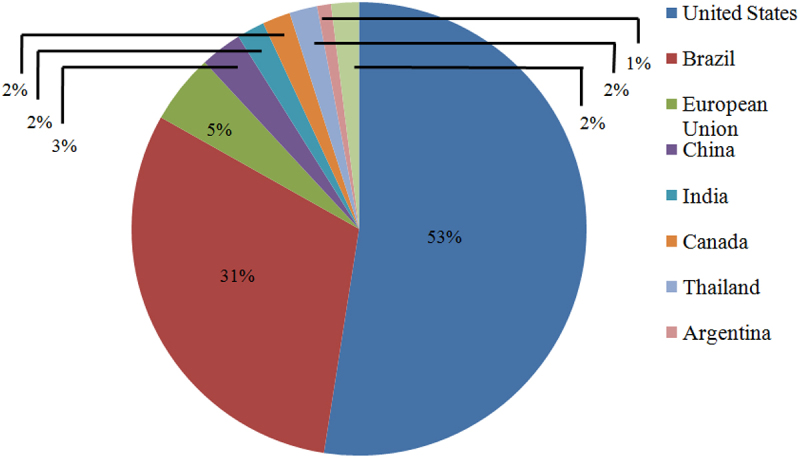
Global annual ethanol production (2021).(Source: Renewable Fuel Association, 2021)

Many countries (developed or developing) have introduced ethanol fuel programmes to promote its usage. According to Renewable Fuel Association [13], Brazil and US are the chief contributors to fuel ethanol, contributing about 31% (8.08 billion gallons) and 53% (13.9 billion gallons), respectively, in 2020. However, due to the COVID-19 crisis, total production has decreased over the past seven years to around 2.9 billion gallons, even below from 2019. A worldwide scenario of bioethanol production in different countriesis represented in Figure 1.

**Brazil**: It has been the largest producer globally for past few decades, however, in 2006, the United States took the place of the top producer of ethanol. It is the first country to generate ethanol commercially through the *Proalcool* programme, which was started by its government in 1975 to utilize it as a substitute for gasoline, considering the increased oil prices. Current , Brazil is the 2^nd^ highest ethanol producer. Its entire ethanol generation in 2020 was 31.35 billion liters, which showed a 16% decrease, as compared to the revised figure, i.e. 37.38 billion liters for 2019 due to the diversion of sugarcane use more toward production of sugars. Fuel ethanol production in 2020 was estimated at 28.66 billion liters, which also showed a 17% decline from the previous year. Corn-based ethanol production was reported at 2.5 billion liters, a rise of 1.17 million liters in contrast to the revised figure for 2019 as per The Brazilian Corn Ethanol Union [17]. While ethanol from cellulosic biomass was expected at around 32 million liters in 2020 and 30 million liters in 2019. In Brazil along 360 units of ethanol mills are operation for ethanol production in 2020 [18].

**United States (U.S)**: It is the most prominent ethanol producer and has 209 ethanol plants located in 25 different states. The United States has encouraged biofuel production over the past few years. Its Energy Policy Act of 1978 established a subsidy for ethanol production and, likewise reduced the federal tax for ethanol-blended gasoline for its implementation [19]. The Energy Policy Act, 2005 also provides economic incentives, tax credits, and loans to promote biofuels and established the Renewable Fuel Standard (RFS) to mandate the 7.5 billion gallons of renewable fuels blending with gasoline by 2012. Energy Independence and Security Act expanded the target of RFS, 2007 (EISA) to generate 36 billion gallons of biofuel by 2022, out of which 21 billion gallons were produced from advanced fuels [20]. In 2020, the U.S. produced about 13,800 million gallons of ethanol, which was lesser from the targetted quantity in 2019. Moreover, total ethanol export was estimated to be 1.3 billion gallons, which declined 9% from the target fixed in 2019.

**European Union (EU)**: The main feedstock used for bioethanol production in the EU is sugar beet derivatives and grains. As per the 2019 biannual progress report, the EU are on the right track to meeting the 20% target and also probable to catch up the goal of 10% usage of inexhaustible energy in the transport sector, which is to some extent facilitated by the ‘double counting’ criteria of biofuels and electricity. In 2018, the EU adopted Renewable Energy Directive II (RED II), which became effective in January 2021, and targetted of 32% renewable energy by 2030 and 14% for the transportation sector. However, the profit of ethanol producers declined in 2019, because of the enhanced cost of feedstock and competition from imported U.S ethanol, which overall reduced by 5.6% of ethanol production. The EU generated 4,680 million liters of bioethanol in 2020, representing a 9.8% reduction due to COVID-19 crisis. The total cereal volume needed for production was estimated around 10.6 MMT, which registered a decline of 1.1 MMT in contrast to projections made in 2019, which accounted for only 3.5% of the total cereal production. Apart from the bioethanol production, distillers dried grains (DDG), yeast concentrates, and wheat gluten are the co-products of the production process. Their maximum theoretical production is expected to reach up to 3.3 MMT, a reduced production of approximately 0.35 MMT from 2019 [21].

**China**: It is the fourth-biggest producer and consumer of ethanol after the U.S, Brazil, and the EU. In 2020, China produced 860 million gallons of bioethanol, contributing 3% to the global bioethanol production [13]. Moreover, China produced various ethanol based products on a large scale, including potable alcohol, industrial chemicals, and fuel ethanol. Dissimilar to other countries, China’s ethanol market was focussed to produce industrial chemicals rather than fuel ethanol. The import of fuel ethanol was restricted till 2015 and also did not produce sufficient ethanol to export. To expand the ethanol market, China’s government provides subsidies on corn processing for ethanol production and nationwide increases the blending of ethanol and gasoline by implementing E10. However, ethanol market of China is still facing challenges like feedstock supply to meet the E10 goal. In 2018, it was reported that 87% of fuel ethanol in China was produced from corn-based feedstock, 11% from cassava-based and sugar cane, and 2% from cellulosic feedstocks. Due to government directives (municipal and provincial), fuel ethanol consumption was estimated to be 4,311 million liters in 2019, which was higher from 2018 (1.397 million liters). In China’s supply for transportation fuel, all the ethanol (fuel) should meet the standard GB18350 for denatured fuel ethanol [22].

**India**: The National Biofuel Policy, 2018 has envisaged target for 20% blending of ethanol in gasoline by 2030, and the Ethanol Blending Program (EBP) demands the ethanol acquisition from sugarcane juice, B and C-heavy molasses, and damaged food grains like broken rice and wheat. In 2019, India also authorized the utilization of surplus rice for fuel ethanol manufacturing. In 2020, the ethanol blending rate has reached 5.2%, which was higher than the blending rate of 2019, i.e. 4.5%. This increase is not due to an increase in ethanol supply but somewhat due to less gasoline consumption (near about 12% low) during the nationwide COVID-19 lockdown.

India has a total of 330 distilleries that can produce over 6 billion liters of rectified spirit annually. Out of which 200 distilleries (approximately) can produce about 3.5 billion liters of ethanol that can be used for industrial, medical-grade, fuel, and potable liquor applications. In 2020, a total of 1.7 billion liters of contracted supplies of ethanol were reported by the Indian Mills Association, which includes 781 million liters, 685 million liters, 111 million liters, and 125 million liters of ethanol produced from C-heavy molasses, B-heavy molasses, damaged food grains, and sugarcane juice, respectively. In 2019, 2.5 billion liters of ethanol were obtained from molasses, and 1.9 billion liters were blended with petrol to get a blending rate of 4.5% [23].

## 3. Bioethanol and sustainable development goals

**Figure 2.**
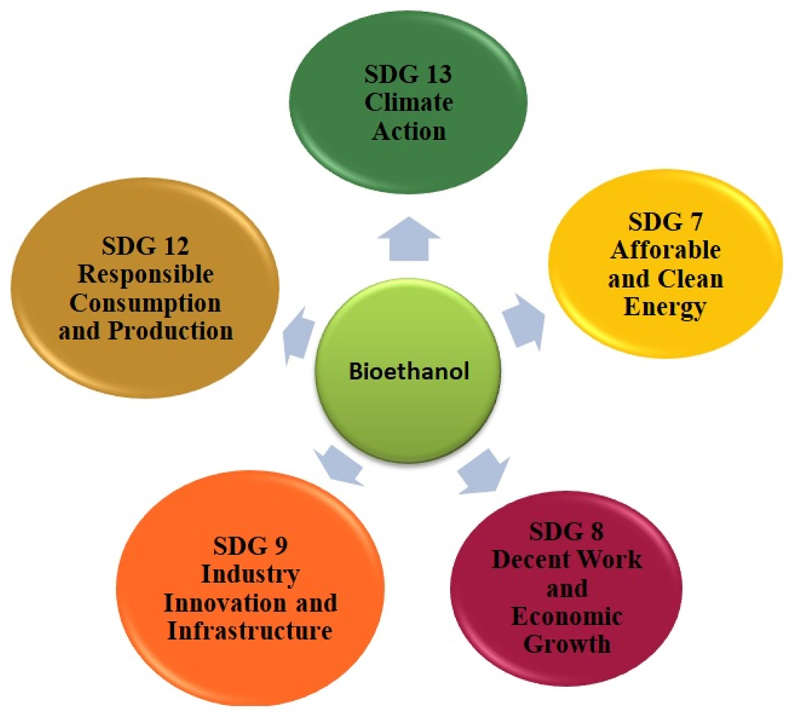
Sustainable development goals and their attibution by bioethanol industry.

The sustainable development goal, affordable and clean energy (SDG 7), includes a substantial rise in the renewable share in the global energy blend by 2030; the global energy mix includes electricity, cooking, heating, and transportation fuel [24]. Although the share of renewables in transportation has been growing since 2008 in several countries. However, it is still less than 4%, with the majority of consumption in the form of biofuels (91%) driven by the support policies of the nation’s [25]. Biofuel demand has grown at an average rate of 5% from 2010 to 2019, but considering the target to achieve Net Zero-emission by 2050, a 14% growth per year is required up to 2030 [26]. Biofuels hold the potential to enhance renewables share in the transportation and energy sector globally, provided that large-scale sustainable production becomes feasible. Apart from SDG 7, biofuels contribute in attaining SDG 13 (climate action) [27]. Bioethanol as a transportation fuel can significantly decrease carbon emissions in the transportation sector. Moreover, the industry can open new job opportunities ranging from building and operating production units for collecting and transporting feedstock [28] under SDG 8 (decent work and economic growth). The second generation of bioethanol uses biomass such as agricultural and forestry residue as feedstock, promoting recycling and encouraging efficient use of natural resources. Biomass is available in an abundant quantity in various countries of the world; therefore, second-generation biofuels could increase domestic consumption and reduce material outsourcing, which agrees with the goal of responsible consumption and production (SDG 12) [29,30]. The bioethanol industry has a vast potential to increase the contribution of manufacturing sector to the country’s GDP and also plays a vital role in generating employment and reduced CO_2_ emissions per unit of economic value (SDG 9- Industry, Innovation, and Infrastructure). Figure 2 depicts the positive impact and contribution of bioethanol industry in achieving different sustainable development goals.

## 4. Generation of biofuels

**Figure 3.**
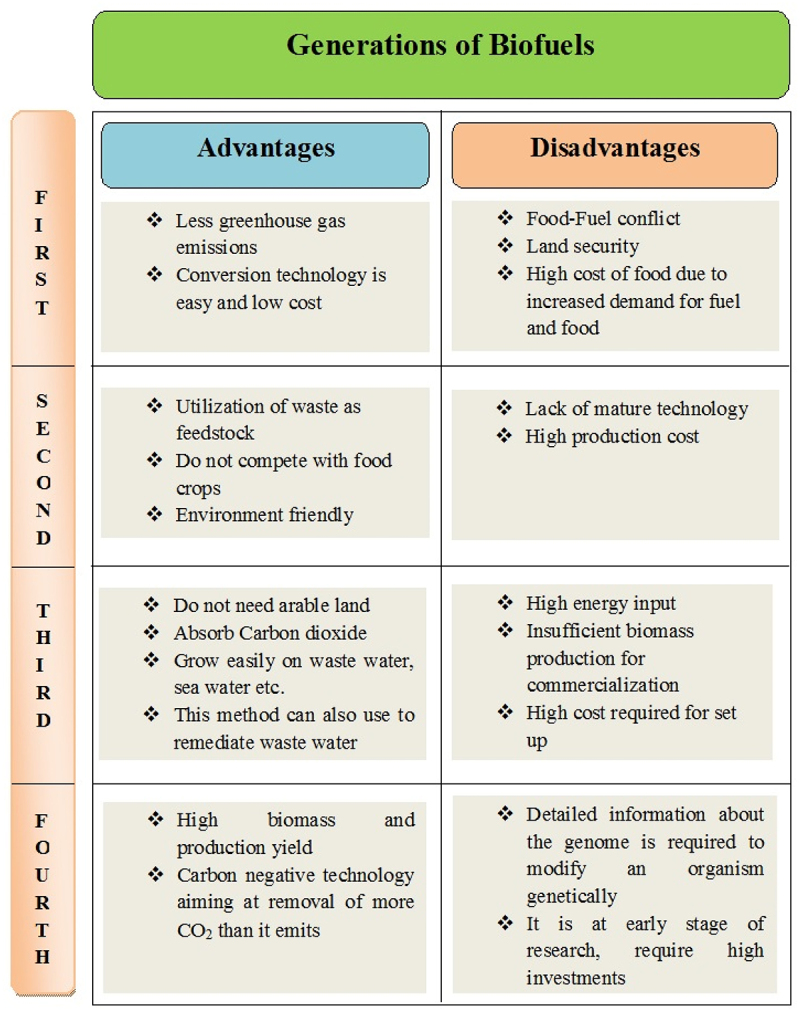
Advantages and Disadvantages of different generations of biofuels.

Biofuel is categorized into four generations based on the feedstock used for its production and its advantages and disadvantages (Figure 3).

### 4.1. First-generation biofuel

This generation is primarily generated from food crops containing a good amount of starch and sugar. Both sugary (sugarcane, sweet sorghum, sugar beet, etc.) and starchy (corn, cassava, wheat, etc.) feedstocks are exercised to produce biofuels. These two feedstocks are converted to biofuels through fermentation, however, the starchy raw material needs to undergo saccharification before fermentation. These materials contain long chains of glucose that must be shortened prior to their efficient transformation into biofuels [31]. This commercialized technology contributes 50 billion liters annually to the total biofuel production [32]. Despite mature technology, it still has some socio-economic and environmental impacts. This technology contends with food for arable land and water resources that might cause soil and land deterioration due to over-fertilization. It can also raise the cost of cereals, wheat, corn, etc [33].

### 4.2. Second-generation biofuel

Second-generation feedstocks made their place in the biofuel production process to avoid food security as raised by first-generation feedstocks. This technology is based on lignocellulosic biomass (LCB) derived from non-edible plants (switchgrass, jatropha, etc.) and leftover agricultural residues (husk, leaves, stems, straw, etc.). LCB is a cheaper carbon source and is readily available in high quantities. Moreover, it is a renewable and environmentally friendly energy resource that has reduced CO_2_ emissions. The biochemical structure of lignocellulosic biomass is complex and is comprised of cellulose, hemicellulose, and lignin. Cellulose and hemicellulose are the polymeric chains of hexose and pentose sugars entangled through covalent bonds. Still, lignin is made of phenyl propane units and behaves like a physical barrier surrounding cellulose and hemicellulose. Lignin must be eliminated prior to conversion of biomass to biofuel [6], which can be achieved by pre-treatment of the biomas. However, this step increases the production cost and produces toxic compounds that reduce the efficacy of the process. To convert this biomass to biofuel, matured/efficient conversion method is required to convert these abundant resources [34]. The lack of robust microorganisms for simultaneous conversion of hexose and pentose sugars into ethanol is another bottleneck in this process.

### 4.3. Third-generation biofuel

Owing to shortcomings of 1^st^ and 2^nd^ generation biofuel, the search for another raw material led to uncovering the algal potential for biofuel production. Biomass from algae for biofuel production is economical and sustainable and can replace fossil fuel demands. Algae are photosynthetic organisms that can produce biomass composed of carbohydrates, lipids, and proteins by utilizing sunlight and CO_2_ [35]. The advantages of using algae as feedstock are- low carbon input, not requiring arable land, and adaptive to growth in wastewater, brackish water, and seawater. This method is promising as it gives a good amount of oil, lipids, and carbohydrates, which can be converted to biofuels. Still, the drying of algal biomass and extraction of its components utilize a high energy input, which is one of the limiting factors of this method [36]. Another limiting factor is the insufficient production of biomass for commercialization [37].

### 4.4. Fourth generation biofuel

This technology was put forth to remove the limitation of the three generations of biofuel technologies mentioned above. These are derived from the plant or biomass that are specially engineered, that may have a higher yield of energy or lower barriers to cellulosic breakdown and can grow on non-agricultural land or bodies of water [38]. The 4^th^ generation of biofuel mainly involves using genetically modified algae to get desirable properties to produce biofuels and capture CO_2_ during the production stage [39]. Besides, plants are also genetically modified for reduced recalcitrance and improved ethanol yield [40]. Genetically modified algae produced a high amount of biomass as compared to unaltered. Hence, it is an excellent alternative to fossil fuels, however, very few studies assessed the scale-up cultivation of genetically modified microalgae and their environmental impacts [41].

## 5. Feedstock

**Figure 4.**
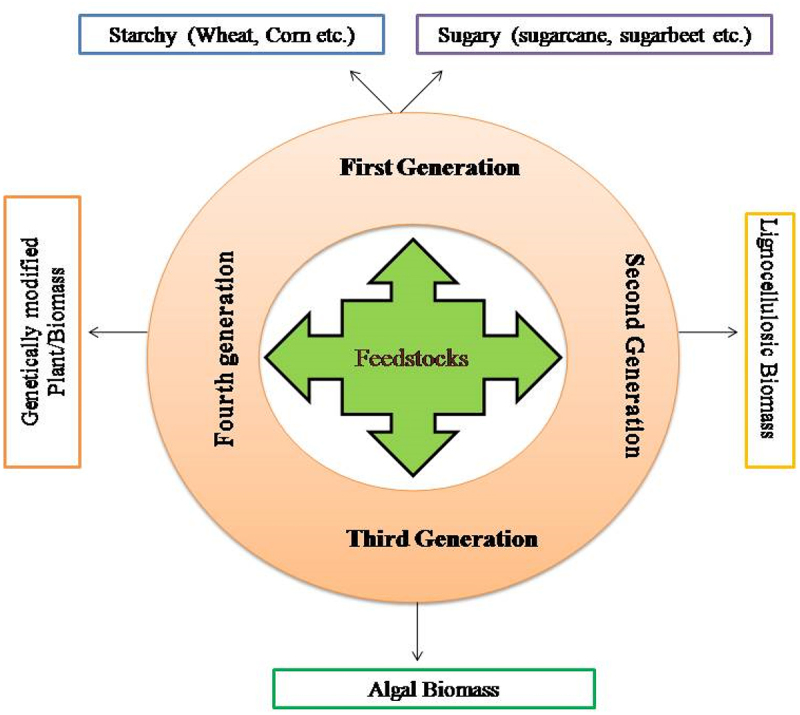
Feedstocks and their contribution in different generations of ethanol production.

The feedstock for the bioethanol production is broadly categorized into four categories, as shown in Figure 4. Different studies on bioethanol production from these feedstocks are mentioned in Table 1.

- First-generation feedstock mainly consists of sugar and starch-containing substances, i.e. sugarcane, sweet sorghum, wheat, corn, etc.
- Second-generation feedstock mainly involves lignocellulosic biomass, i.e. the residual part of crops and non-edible energy crops like wheat straw, rice husk, sugarcane bagasse, etc.
- Third generation feedstock- It includes the algal biomass, i.e. microalgae and macroalgae.
- Fourth-generation feedstock- This generation is not using feedstocks as available in nature, but modified or altered by genetic engineering to improve the raw material features for better results and efficiency.

**Table 1.** Bioethanol production from different feedstocks.

Generation	feedstock	Ethanol production	References
First	*Amorphophallus* sp (starchy tuber)	8.68 ± 0.91 g/L	Bhuyar et al.[42]
First	Sugarbeet pulp	12.6 g/l	Berlowska et al.[43]
Second	Corn stover	34.3 g/l	Liu and Chen [44],
Second	Cassava stems	263 ml/Kg dry biomass	Pooja et al.[45]
Second	Cassava leaves	200 ml/Kg dry biomass	Pooja et al. [45]
Second	Cassava peels	303 ml/Kg dry biomass	Pooja et al. [45]
Second	Corn cobs	16.928 g/L	Laltha et al. [46]
Second	Sorghum biomass	36 g/l	Joy and Krishnan [47],
Second	Potato peels	1.513 g/L/h	Chohan et al. [48]
Third	Desmodesmus specie	4200 l/ha/year	Smachetti et al. [49]
Third	*Eucheuma Denticulatum Spinosum macroalga*	11.6 g/g	Alfonsín et al. [50]
Third	*Ulva intestinalis*	0.081 g/g dry weight	Osman et al. [51]

### 5.1. First-generation feedstock

#### 5.1.1. Sugar containing raw material

The countries like Brazil, Germany, France, and India mainly produce bioethanol from sugar-containing energy crops that include sugarcane, sugar beet, and sweet sorghum, with yields of 62–74 tons/ha, 54–111 tons/ha, and 50–62 tons/ha, respectively [52]. Production from first-generation feedstock is a high sugar yield with production cost. But seasonal variability of these raw materials restricts their use. Some potential sugar-based raw material has been discussed below:

**Sugarcane**: It is a C4 plant that efficiently converts solar radiation to biomass grown in tropical and subtropical regions [53]. For many years, sugar cane juice and molasses have been utilized in bioethanol production. In Brazil, bioethanol production from sugarcane contributes about 79%, and molasses are India’s top raw material [54]. Brazil used the Melle-Boinot (MB) bioethanol production from sugarcane. Brazil bags first in producing sugarcane with 7681 million tons followed by India with production of 341.2 million tons [55].

**Sugar beet**: It is grown in temperate zones with low rain rainfall and produces 20–25 tons per acre. The feedstock has been used for biofuel production in Europe, North America, and France. Ethanol from sugar beet has also been experimented with in tropical countries like China, Kenya, the USA, Brazil, and Australia [56]. Freshly harvested sugar beetroot is composed of water (75–76%), sugars (15–20%), non- sugars (2.6%), and pulp (4–6%). Processing 1 ton of sugar beetroot yields 121 Kg of sugar, 50 Kg pulp, and 38 per 1000 g of molasses that contain 18.2 per 1000 g of sugar, 7.8 per 1000 g of water, and 12.1 per 1000 g of impurities [57]. The expense of ethanol production from sugar beet is more than from sugarcane because of the significant amount of chemicals and energy input [58]. Ethanol production of 12.6 g/l was obtained from carbohydrates liberated from sugar beet pulp by co-culture of *Saccharomyces cerevisiae* Ethanol Red, and *Scheffersomyces stipitis* LOCK0047 [43].

**Sweet sorghum**: It is also a C4 photosynthetic crop that is highly adaptive and resistant to drought, salinity, waterlogging, and acid toxicity. It contains high sugar content in its stalk and produces 20 gallons of ethanol from 1 ton of stalk on an average [58]. The extracted juice from sweet sorghum contains about 16–23% Brix [55]. It can grow as a perennial crop in both regions, i.e. temperate and tropical. The US Department of Agriculture (USDA) has reported that during the biofuel extraction from sweet sorghum, the energy input and the energy output ratio is 1:8 [59]. Ethanol production from sweet sorghum is environmentally friendly as it has low sulfur content, less BOD and COD, and high-octane ratings. It can also potentially produce ethanol up to 8000 l/ha/year, which is double to the potential of corn [60]. Erasmus et al. [61] evaluated the sugar and ethanol yield from 9 sweet sorghum genotypes in the Adana location, resulting in total sugar content between 1245 and 5909 kg/ha and ethanol yield between 738 and 3146 L/ha.

#### 5.1.2. Starch-based feedstock

Starch consists of many glucose monomers and is extensively utilized for bioethanol because of its easy availability and conversion, long storage duration, and high ethanol yield [62]. Starch containing feedstock includes grain crops, i.e. corn, wheat, barley, and tuber crops, i.e. cassava, potato, and sweet potato. The starch must be hydrolyzed before fermentation to break the long chains of carbohydrates for ethanol production. A few starchy feedstocks have been discussed below:

**Corn**: It is a crucial starchy crop that is widely used for bioethanol production. A tremendous amount of corn is generated in North America, Asia, Europe, and South America [63]. Ethanol production from corn is based on its variety and quality. Based on corn variability, ethanol yield ranges from 3 to 23% [64]. The USA produced ethanol from corn and nearly produced 13.8 billion gallons of ethanol in 2020, representing a 12.7% decrease from the previous year [65]. Chen et al. [66] integrated corn with corn stover for better ethanol titers. Alkali pretreated corn stover hydrolyzate was mixed with the liquefied corn for ethanol production, resulting in ethanol yield and productivity of 80.47% and 2.19 g/l/h, respectively.

**Wheat**: It is available globally, mainly in Asian and European countries. Global production of wheat in 2020-2021 was 775 million metric tons, and China, being the primary producer of wheat, produced 137 million metric tons in 2020-2021 [67,68]. It has also been reported that 1 MJ of wheat-based ethanol consumption can diminish GHG emissions by 42.5–61.25% as compared to gasoline [69]. At industrial scale technology, one wheat bushel can produce nearly 2.6 ethanol gallons which is relatively less than corn-based ethanol (2.8 gallons of ethanol from 1 corn bushel) [70]. Mikulski and Kłosowski [71], utilized microwave-acid pretreated wheat and rye stillage for ethanol production. The substrate was then hydrolyzed with Cellic® CTec2 with a dose of 1.0 FPU per 1 g of wheat and rye stillage dry weight. An ethanol concentration of 20 g/l was obtained after 48 h of the fermentation time.

**Cassava**: It is amongst the potential tuber crops available for ethanol generation and can survive in tropical and temperate climates. It can tolerate a semi-arid environment with less than 500 mm of rainfall. The cassava market was at 6.90 million MT in 2019 globally, and Thailand was the largest producer in the world [72]. Its use for production receives attention due to its high starch yield and capability to resist heat and drought and grow on degraded land [73]. Pradywong et al. [73] compared the cassava starch with corn starch utilizing the conventional and granular hydrolyzing method. The ethanol concentration was 2.8% higher in the granular hydrolyzing method than in the conventional one. Overall, cassava’s fermentation and ethanol profiles are somewhat similar to corn, making it an attractive option for bioethanol production.

### 5.2. Second-generation feedstock

There is no food security issue related to second-generation feedstock as it is comprised of non-edible biomaterial. Niju et al. [74] reported that 50% of the biomass produced in the world is lignocellulosic, which has energy potential higher than that of required world primary energy demand. The feedstock involves residues of food crops, non-edible crops, and lignocellulosic biomass from other industrial and urban sources. It is a carbon reservoir mainly comprised of three main constituents, i.e. cellulose, hemicellulose, and lignin. Cellulose is a polysaccharide made of straight chains of C_6_H_12_O_6_ units connected by β-1, 4 glycosidic bonds represented by a formula (C_6_H_10_O_5_)_n_; where, n is the degree of polymerization (DP) [75]. It has a complex physical structure because free OH groups present on C2, C3, and C6 form intra and inter hydrogen bonds. This interaction forms cellulose chain aggregates, leading to bundle formation called microfibrils. The assemblage of microfibrils forms macrofibrils which in turn form cellulose fibrils. This tight adherence made the rigid cellulose structure with a high melting point than required for chemical degradation [76]. Cellulose has two regions- crystalline and amorphous. The crystalline region is well arranged and compact, while the amorphous part is not much orderly arranged. Therefore, amorphous part is more susceptible to degradation, while the crystalline part poses difficulty in degradation [77]. Hemicellulose is a branched heteropolymer that mainly consists of two sugars, i.e. pentoses (xylose and arabinose) and hexoses (glucose, galactose, mannose, and rhamnose). The structure of hemicellulose is much different from cellulose in context to the small number of building blocks made of 500 to 3000 repeated sugars fractions that form various short chains. In contrast, cellulose is made of 7000–15000 glucose units [6]. It interlinks with cellulose and lignin to create a network structure of plant cell walls. Hemicellulose utilization is tricky due to presence of variable sugars, as pentose hydrolysis is not very simple [78]. On other hand, lignin is a branched and amorphous structure with phenylpropane units associated with a three-dimensional structure [79]. Three primary phenylpropane units are involved in forming lignin structure, i.e. guaiacyl, syringyl and coniferyl. It works as a binding agent that binds the cellulose fibrils and makes its accessibility difficult. Therefore, lignin conversion or removal is necessary to get the ethanol from lignocellulosic biomass [74]. Several potent second-generation feedstocks are available worldwide and used for bioethanol production. Some of them are mentioned below.

**Wheat straw**: It is produced abundantly worldwide with a lignocellulosic content of 33–40% cellulose, 20–25% hemicellulose, and 15–20% (w/w) lignin. Various countries like India, the USA, China, European Union, and Canada are involved in wheat cultivation and annually produce approximately 850 million metric tons of wheat straw [80]. The estimated data have revealed that the 354Tg of wheat straw can produce 104 GL bioethanol [75]. Ziaei-Rad et al. [81] fermented ionic liquid (IL)-pretreated wheat straw hydrolyzate with *S. cerevisiae* resulted in 43.1 g/l of the ethanol production after 48 h fermentation period. Yuan et al. [82]developed a method to convert wheat straw into bioethanol, silica and lignin. Silica was extracted by treated wheat straw with 0.2 mol/l NaOH at 30ºC on incubating for 5 h resulting in 91% of the extraction. Further, the ethanol concentration of 31.1 g/l was produced by *S. cerevisiae* from a hydrogen peroxide pretreated substrate.

**Rice straw**: The annual global production of rice is 750 million metric tons, contributed by more than 50 countries. The continents mainly involved in rice straw distribution are Africa, Asia, America, and Europe. As a result, rice producing countries contribute a large amount of agricultural rice residue, i.e. about 91% of the total production [83]. The cellulose and hemicellulose part of rice straw shared to more than half of the total percentage and can be converted into bioethanol [84]. Further, it has been estimated that straw amounting 668 million metric tons can theoretically produce 282 billion liters of ethanol with suitable technology [80]. Anu et al. [85] optimized the process for chemical pretreatment of rice straw for bioethanol production. Rice straw was pretreated with NaHCO_3_, CaCO_3_ (0.5, 1.0, 2.5 and 5% w/v) and NH_3_ at different concentration of 5, 10, and 20% v/v at 121 °C, 15 psi for a period of 1 hour. Among the three pretreatment agents, treatment with ammonia released maximum reducing sugars. The optimal 10% concentration of ammonia generated 233.76 ± 1.23 mg/g yield of reducing sugars after enzymatic saccharification of 6 hours. Fermentation of ammonia pretreated rice straw with ammonia pretreated enzymatic hydrolyzate was fermented by *S.cerevisiae* at 30ºC, pH 7.0, 150 rpm. Moreover, the whole setup was able to achieve yield of 24.37 g/L bioethanol after 72 h with 20% hydrolyzate. Jin et al. [86] also reported that hydrolysis of NaOH (1%) pretreated rice straw with 200 FPU/ml crude enzyme liberates 22.15 g/l reducing sugar in 20 h. The ethanol yield of 83.5% was obtained in the separate hydrolysis and fermentation process by *Saccharomyces tanninophilus*.

**Sugarcane bagasse**: The sugarcane bagasse contains cellulose (40–45%), hemicellulose (30–35%), lignin (20–30%), and a low amount of ash (1.9%). Less ash content makes it better than the feedstocks like wheat, and rice straw, which contains 9.2% and 14.5% of ash content, respectively [52]. Around 250 to 280 kilograms of bagasse was produced from each ton of sugarcane [87]. Its immediate presence in the biorefinery of sugar extraction is advantageous because the integrated biorefinery approach provides higher rate of ethanol production as compared to individual 1^st^ and 2^nd^ generation plants [88]. An estimated quantity of sugarcane, i.e. 1.6 billion tons from the sugar industry, produced about 493 million metric tons of bagasse annually at a global level [89]. Jugwanth et al. [90] valorize sugarcane bagasse for ethanol production through simultaneous saccharification and fermentation. The highest ethanol concentration of 4.88 g/l was achieved under optimal conditions of 39 ºC and 100 U/g enzyme loading.

***Miscanthus***: Miscanthus is a perennial C4 tall grass that grows faster and provides higher yields without much input of water and fertilizers. Around 20 species of *Miscanthus* are well known, especially in tropical and subtropical regions [91]. It is a potential energy crop that contains about 76.20 to 82.76% of holocellulose and 9.23 to 12.58% of lignin content based on the type of species and genotypes of *Miscanthus* [92]. The potential of bioethanol production from *Miscanthus sacchariflorus* was evaluated by Kang et al. [93] at a bench-scale plant and reported production of one lakh kiloliters of ethanol from 606,061 tons of *M. sacchariflorus.*

**Switchgrass**: Switchgrass is a herbaceous and perennial grass, indigenous to North America, which can be used to produce ethanol. Marginal lands can be utilised for their cultivation and moreover, it can help in conservation of croplands. It can be grown on wide varieties of soil and can survive under variable environmental conditions [75]. Zhang et al. [94] has revealed that about 59 million ha of China’s marginal lands are suitable for switchgrass cultivation and able to produce 22 million tons of ethanol. The estimate also reckoned that the use of switchgrass for bioethanol production can also help to diminish 94% of the greenhouse gas emission compared to gasoline [95]. Dien et al. [96] pelletized the switchgrass to compare the pellets and non-pelletized substrate. Glucan and xylan content were reduced during pelletization, however, glucose, total sugar yield and ethanol yield were not affected.

### 5.3. Third-generation feedstock

The 3G feedstock constitutes mainly the algal biomass. It is an appropriate substrate for ethanol production by its significant lipid and carbohydrate content and ability to grow in variable aquatic conditions. With its ubiquitous nature, the photosynthetic organism represents a broad group with several species that vary in size, morphology, occurrence, and physiology [97]. Based on morphology, algae can be classified into two main types- microalgae (unicellular) and macroalgae (multicellular). Microalgae are single-celled photosynthetic organisms mainly composed of proteins (30–50%), carbohydrates (20–40%), and lipids (8–15%) [98]. Microalgae is classified into five categories including, *Bacillariophyceae, Chlorophyceae, Cyanophyceae, Chrysophyceae*, and *Rhodophyceae*. About 50,000 species of microalgae are estimated, but only a few were used practically [99]. In contrast, macroalgae are multicellular marine organisms containing a high amount of carbohydrates, i.e. near about 60% and 20–30% starch [100]. It is grouped into three classes based on the pigmentation present, i.e. *Phaeophyceae*, *Rhodophyceae*, and *Chlorophyceae*. There are about 9200 species of macroalgae, of which 221 are economically significant [101]. Both microalgae and macroalgae are potent feedstock for bioethanol production. The macroalgae has advantage over microalgae with its lower lipid content and higher sugars and carbohydrates content. According to projections made by the United States Renewable Fuel Standard, the country will achieve target of 36 billion gallons of biofuel production from microalgae fuels by 2022 [102]. Many enterprises, such as Sapphire Energy, Algenol, Seambiotic, etc., produce bioethanol at a large scale, with an annual production of 1 billion gallons, costing only 85 cents per liter [103]. *Eucheuma Denticulatum Spinosum* macroalga was selected as a substrate for ethanol production by Alfonsín et al. [50]; on treating with different concentrations of sulfuric acid, i.e. 3, 5, 7 and 9 % (w/w) in the ratio of 1:7, 1:10 and 1:13 (w/v) at different time intervals (35, 70 and 105 minutes). The hydrolyzate was fermented by *S. cerevisiae*, providing 11.6% yield of ethanol at 9% (w/w), 1:7 ratio and 70 min reaction time. Sulfahri et al. [104] pretreated the marine algae with fungi with an inoculum size of 5 to 20% (v/v) at 30 ºC for 0–120 h before enzymatic hydrolysis. Fungal pretreatment before hydrolysis increased its sugar yield by 2.3 fold. Phwan et al. [105] assessed the impact of pretreatment on bioethanol production from microalgae. The algal biomass was pretreated with dilute sulfuric acid and acetic acid at different concentrations of acids (1, 3, 5, 7 and 9% v/v). The ethanol yields, i.e. 0.28 g/g and 0.23 g/g, were obtained at 5% (v/v) sulfuric acid and acetic acid, respectively.

### 5.4. Fourth-generation feedstock

The fourth-generation feedstock for bioethanol includes algae and biomass crops engineered to augment more carbon than the unaltered species [31,106], making them carbon neutral and a negative carbon source of fuel. *Chlamydomonas, Phaeodactylum, Synechococcus*, and *Nannochloropsis* are some microalgae species genetically modified for enhanced biomass and reduced nutrient consumption [41]. Genetic modifications are employed to reduce the recalcitrance of plant biomass to pretreatment, which increases sugar yield and subsequent ethanol conversion. Modifying switchgrass for reduced lignin content increases the bioethanol yield significantly using the conventional fermentation process [107]. Similarly, plants are engineered to produce cellulase enzymes themselves which reduce the recalcitrance of cell walls and make them amenable to pretreatment and hydrolysis [108]. Apart from this feedstock, genetically modified cyanobacteria are used to directly convert CO_2_ into ethanol [109]. Fasahati et al. [110] designed a process in which biodiesel is co-produced with ethanol from genetically modified lipid producing sorghum. It has been evaluated that the coproduction of biodiesel lowers the minimum selling price of ethanol from $3.08/gal to $2.46/gal.

## 6. Bioethanol production processes

The bioethanol production process has three unavoidable steps: (a) obtaining fermentable sugars, (b) sugar to ethanol conversion (c) ethanol separation and its purification. The process steps of ethanol production from different substrates are illustrated in Table 2. One or more steps can be combined, however, these steps depend on the feedstock and the conversion technology.

**Table 2.** Process steps of generations of bioethanol.

Process	1^st^ generation	2^nd^ Generation	3^rd^ Generation
Feedstock	Starchy and sugary	Lignocellulosic biomass	Algal biomass (cultivation and harvesting)
Pretreatment	Grinding/Milling	Physical, chemical, physicochemical and biological	Physical, chemical, physicochemical and biological (to break the cell structure)
Hydrolysis	Enzymatic hydrolysis (α-amylase and glucoamylase)	Acid and enzymatic hydrolysis (cellulases, hemicellulases)	Enzymatic hydrolysis (Cellulases, α-amylase, glucoamylase and β-glycosidase)
Fermentation	SHF, SSF with the help of yeast strain		

### 6.1. 1^st^ generation (1G) bioethanol production

It is usually produced from sugar or starch-based substrates by fermenting the extracted sugars of the feedstock. Depending on the feedstock, i.e. sugar or starch-based, the processes differ from each other. Despite the variation in the process conditions, some main stages remain same [111]. USA and Brazil are the two major bioethanol contributors, which uses corn and sugarcane as significant substrates for ethanol production. The technology used for 1G bioethanol from corn and sugarcane is discussed below.

#### 6.1.1. Starch-based feedstock

Ethanol cannot be directly produced from starch because of its long-chain polymeric structure. Hydrolysis is necessary to convert the polymer into monomers. In general, few steps needs to be followed for bioconversion of starch to ethanol, including milling, liquefaction, hydrolysis, fermentation, and distillation [112]. Corn-derived bioethanol can be generated using two main techniques i.e.drying and wet milling. The corn is first grounded in dry milling and then mixed with water to produce a slurry. In later stages, α- amylase is added to the grounded corn slurry to make amylase and amylopectin susceptible to the hydrolysis process and converted to dextrose and maltose sugars.

Further, the addition of glucoamylase hydrolyzes the maltose to glucose at a temperature of 60 ºC for about 2 hours under continuous agitation. The extracted sugar is futher fermented on Inoculating with different strain of yeast to produce bioethanol. A distillation step is also necessary to segregate the ethanol from the solid fraction. After ethanol recovery, the residual part can be used as animal feed [113]. More than 90% of fuel ethanol in the US is produced from the dry mill process.

In the wet milling process, the corn is first subjected to the dilute sulfur dioxide solution for 24 to 48 hours for its decomposition. With the help of various grinders (hydrocyclones, continuous centrifuges), corn germ is separated from the solution, and the oil-rich corn is used as a by-product. A grit screen is used to set apart the starch, gluten, and fiber. Finally, the starch-rich part isconverted to bioethanol, on following the steps similar to dry milling process [114].

#### 6.1.2. Sugar-containing feedstocks

The process steps of bioethanol production from sugar-based substrates do not require the liquefaction and hydrolysis stage in contrast to the starch-based bioethanol process. The first step is milling, which includes sugarcane washing, chopping, and shredding. The milling process is followed by juice extraction with the help of crushers, chemical treatment to remove impurities, concentrate the saccharose sugar, and ultimately sugar fermentation to get bioethanol. The waste by-product bagasse is generated and applied as raw material for the 2G bioethanol refinery. The whole methodology for bioethanol production from sugarcane is called as Melle- Boinot process. After distillation, ethanol is recovered, and vinasse (the nonalcoholic part) can be used as a fertilizer due to its richness of nutrients like nitrogen, potassium, calcium, etc [115].

### 6.2. 2^nd^ generation (2G) bioethanol production

Bioethanol production from biomass, which is also known as 2^nd^ generation bioethanol, mainly includes a pretreatment, hydrolysis, and fermentation.

**Pre-treatment**: The first step of the 2G bioethanol process includes the biomass pretreatment that disrupts the lignocellulosic biomass complex. The main goal of pretreatment is to decrease the cellulose crystallinity and remove lignin, in order to make the biomass accessible to enzymes in hydrolysis steps. The pretreatment is divided into four main kinds- physical, chemical, physicochemical, and biological. The physical pretreatment method is mainly applied to diminish the biomass size and enlarge the concerned biomass’s surface area. Grinding, milling, chopping, freezing, and extrusion are ways through which physical pretreatment can be attained [116]. The chemical pretreatment method involves use of chemicals, i.e. acids, alkali, other organic solvents, etc. The acid utilization for pretreatment of biomass can solubilize the polysaccharides into monosaccharides. Internal bonds between the lignin and hemicellulose have been broken down using alkali pretreatment [2]. The physicochemical method involves the combined effect of the physical and chemical methods. It encompasses steam explosion, ammonia pretreatment, wet oxidation, carbon dioxide explosion, and liquid hot water pretreatment. In the steam explosion method, the chipped biomass is subjected to high-pressure steam that changes the lignin structure and decomposes the hemicellulose, improving cellulose hydrolysis. Like a steam explosion, ammonia fiber pretreatment and carbon dioxide explosion incorporate the biomass subjection to liquid ammonia and CO_2_, respectively, at elevated temperature and pressure for a short duration, thereby enhancing the cellulose digestibility [117]. Biological pretreatment entails using microorganisms or enzymes to disintegrate the lignin and hemicellulose. This method consumes limited energy but is time-consuming [34]. Sometimes to enhance the efficiency of the process, two or more pretreatment methods are combined to get desired results.

**Hydrolysis**: This step is aimed to achieve fermentable sugars from the cellulose and hemicellulose polymers. Usually, hydrolysis can be achieved by acid or enzyme utilization. Acid hydrolysis is of two types i.e. dilute and concentrated. Dilute acid hydrolysis occurs at high temperature and pressure conditions with low acid concentration for a few seconds to minutes, while concentrated acid hydrolysis utilizes high acid concentration at low temperature and pressure. It can remove lignin and decompose the cellulose and hemicellulose to get fermentable sugars. The dilute acid hydrolysis works at high temperatures that generate inhibitory compounds like furan and phenolic compounds, carboxylic acids, etc., on hemicellulose degradation. The inhibitory compound generation by acid hydrolysis affects the efficiency of ethanol production, therefore, it becomes one of the disadvantages of this process. The other drawback of this technique is the corrosion problem that occurred due to the use of acid [118].

In enzymatic hydrolysis, various enzymes are employed to solubilize the polymeric assemblage of lignocellulosic biomass. Cellulase complex is mainly used to hydrolyze cellulose and does not produce inhibitors. It is produced mainly by fungi and classified into three main types, which include endoglucanase (breaks down the amorphous part of cellulose structure), exocellulase or cellobiohydrolase (degrades the reducing and non-reducing ends of the cellulose polymer and produces cellobiose) and β- glucosidase (act on cellobiose to produce glucose) [119]. Due to different sugar units, an enzyme complex, i.e. hemicellulase, is also used to hydrolyze its polymeric parts of hemicellulose. The enzymatic hydrolysis process is particular, and its rate and extent rely on the enzyme loading, hydrolysis time, efficacy of cellulase, and other structural characteristics [120]. The saccharification success also depends on delignification by pretreatment [121]. This method is non-chemical but expensive and time-consuming [122].

**Fermentation**: It is the method of transforming fermentable sugars into bioethanol with the help of microorganisms. It can be done in three distinct configurations, i.e. separated hydrolysis and fermentation (SHF), simultaneous saccharification and fermentation (SSF), and consolidated bioprocessing (CBP). SHF provides a chance to work on optimal conditions of both processes, i.e. hydrolysis and fermentation. But the end product inhibition (glucose accumulation) is the main limitation of this procedure [123]. SSF removes the drawback of sugar aggregation and reduce the number of steps as both (hydrolysis and fermentation) are combined in SSF. But the variation in the required temperature of enzymes and fermenting microorganisms is the drawback of this technology [124]. CBP is the process that unites all the process steps. Reduction in the steps can decrease the capital cost of this technology. Although the process cost is reduced on modifying microbial strains to hydrolyze the biomass and convert it into the end product under similar conditions [125].

### 6.3. 3^rd^ generation (3G) bioethanol production

Algae (microalgae or macroalgae) rich in lipids and carbohydrates and having less lignin are the main reasons to consider it for bioethanol production. The stages of bioethanol generation from algae are discussed below.

**Strain selection**: The foremost step for bioethanol production from algae is the choice of the strain. Several species of algae are present, but not all are suitable for bioethanol production considering the factors including photosynthetic activity, biomass productivity, growth rate, carbohydrate content, and ability to withstand different temperatures and pH conditions. The strain with high carbohydrate content, and less lignin content which can tolerate stressful conditions, is suitable for bioethanol production [101].

**Cultivation**: The growth and cultivation of algae depend on light, salinity, temperature, pH, CO_2_, and availability of macro and micronutrients. These parameters jointly affect the photosynthetic activity responsible for producing macromolecules required for biofuel production. Therefore, providing an optimum condition for good productivity is much essential. Various cultivation strategies like open, closed, and hybrid systems can be utilized for algal cultivation [126]. The open pond system is used commonly across the globe and is shallow with 15–30 cm depth. In an open pond system, location is the main factor as a sufficient amount of sunlight and carbon dioxide is required for algal growth. The open pond system becomes more sustainable if algae are cultivated on wastewater, providing dual benefits, i.e. bioremediation and biofuel production. This system has low maintenance, but its monitoring is the complex probability of contamination, needed more land, and weather constraints [127]. The system mostly applied for research, and industrial algal growth is a raceway pond, circular tank, and a big shallow pond.

Closed systems can overcome the drawbacks of an open system. Photobioreactors (PBRs) are designed to provide more light with less contamination. It is perhaps made as tanks, bags, or towers. PBR could be tubular or plate made of glass or plastic. This method provides more yields but is expensive and requires more energy than the open system [128]. A combined set-up of the open and closed system is referred to as a hybrid system. It is constructed to eliminate the limitations of both systems. In this system, the algae are first grown in a closed photobioreactor and then shifted to an open system to increase the yield. It is appropriate for large-scale algal cultivation [129].

**Harvesting**: Harvesting is the process of separating the algal cells from the medium without creating any damage to the viability and activity of the cell. Many harvesting techniques collect algal biomass, including flocculation, floatation, filtration, centrifugation, and precipitation. The choice of the procedure relies on the cell size and density [130]. Harvesting is followed by dehydration of algal biomass because it contains 90% water content. Therefore, different drying methods, including sun drying, sprig drying, freeze-drying, etc., are engaged, for drying to get solid hard material. However, each technique has its pros and cons. Sun drying is an inexpensive approach but consumes more time and requires a large area for drying. Sprig drying is needed to extract valuable products but causes harm to the pigments of algae and is an outrageous method. The freeze-drying method also dehydrates algae efficiently but is expensive, and its operation is complex on a large scale [131].

**Pre-treatment**: Pretreatment is the necessary step to get bioenergy from algal biomass. The chief target of the algal biomass pretreatment is to disintegrate the cell wall and modify the intracellular compounds. The cellulose is found in the cell wall of the algae and starch in the plastid as a sugar reserve. The cell wall comprises of two layers, i.e. external and internal, while the external layer is a matrix of pectin, agar, alginate, and algae polymer. At the same time, the internal has fucans, hemicellulose, pectin, glycoproteins in the cellulose matrix, and a small amount of xylose, rhamnose, arabinose fucose, and galactose [132]. The rigidity of the cell wall depends on its composition, which varies from species to species. Starch is present in the form of semi-crystalline granulose particles made of amylase polymer and amylopectin. The crystalline granules of starch contain water, making it more stable and harder to hydrolyze through enzymes [133]. Therefore, it is essential to gelatinize it through different pretreatment methods mentioned as physical, chemical, physicochemical, biological, and combined methods [134].

**Hydrolysis and Fermentation**: Enzymatic hydrolysis of algal biomass requires cellulase, amylase, and glucoamylase as it mainly comprises cellulose, starch, and a small amount of hemicellulose. Hydrolysis of cellulose and starch based on the breakdown of β-1,4-glycosidic bonds amid the hydroglucose subunits into cellobiose and cellodextrin, further degraded to glucose by β-glucosidase. The disruption of α-1,4-glycosidic linkages into dextrin on degradation by glucoamylase generates glucose and oligomers, respectively. This method is of low maintenance and does not produce inhibitors, however, enzyme cost can be a drawback for its large-scale production [135]. The released sugar is fermented to ethanol with the help of yeast by using any of the fermenting techniques, i.e. SHF or SSF.

## 7. Factors affecting ethanol production

Different physical and nutritional parameters affect ethanol production, which must be optimized for efficient yield and productivity. Table 3 illustrates the parameters responsible for high and low titers of ethanol.

**Table 3.** Factors affecting ethanol production.

Parameter		Range
Physical	Temperature	20 to 35 ºC (fermentation)
	pH	4 to 8 (depends on the substrate and the strain employed)
	Incubation period	Moderate
	Agitation rate	150–200 RPM
Nutritional	Substrate Concentration	Moderate
	Inoculum size	10^4^ −10^7^ cells/ml
	Nutritional Components	Moderate dose but it may vary according to the selected organism requirement
	Reducing agent	Low concentration

### 7.1. Physical parameters

**Temperature**: It is one of the physical parameters that must regulate meticulously throughout the production process. Temperature effects enzyme activity, microorganism growth, and overall process efficiency [136]. IThe studies in this field elaborated that ethanol concentration elevates with rise in temperature. However, an increase in temperature stressed the growth of the fermenting microorganism and made them to produce heat shock proteins and deactivate their ribosomes. High temperature also denatures the tertiary structure of the enzymes involved in the microbial growth and fermentation process [137].

On the other hand, a lower temperature range might cause the cells’ lower specific growth rate. Therefore, it is essential to optimise temperature during the fermentation process to give maximum productivity and yield. The optimum conditions mainly used for the fermentation process ranges from 20 to 35ºC. Fakruddinet al. [138] reported that ethanol production increases during fermentation temperature of 25–30 ºC and then sharply decreases on further increasing in temperature. The studies also able to achieve an ethanol production rates of 28.84 g/L, 86.9 g/l and 65.33 g/l at 25ºC, 30ºC and 35ºC, respectively and reached to conclusion that 30ºC is the ideal temperature for the production of ethanol by *S. cerevisiae* IFST-072011.

**pH**: The pH of the media broth plays a vital part in the biofuel processes, i.e. hydrolysis and fermentation. A particular pH is required for the enzyme activities like 4–5 for glucoamylase, 5–8 for α- amylase, and 4.5 to 5.5 for cellulase. It also affects the microorganism and their cellular activity directly [64]. Generally, a change in the proton concentration (H^+^) in the media broth can influence the plasma membrane’s total charge, impacting permeability of certain nutrients in the cell [139]. The ideal pH conditions were different based on the substrate and the strain employed in the procedure. For ethanol production, the pH range best suited for *S. cerevisiae* and *Zymomonas mobilis* is 4 to 5 and 5 to 6, respectively [140,141]. Bernardi et al. [142] used a three-factor central composite design to optimize biomass saccharification parameters and enhance the reducing sugar yield. According to the findings of the study, recombinant xylanases exhibited higher levels of activity at 80ºC and a pH range of 4–8.

**Incubation period**: Microorganism growth is greatly affected by fermentation time. A shorter fermentation period is responsible for insufficient growth of microorganisms as it does not get enough time to adapt to the existing conditions, which hampers the efficiency of fermentation process. However, a longer fermentation time also interrupts the microorganisms’ survival as the ethanol present in the broth has toxic effects on the growth of microbes [143]. For effective hydrolysis, enzymes need time to reach the carbohydrate molecules. A shorter duration may result in the incomplete conversion of the carbohydrate polymers due to the interruption in the hydrolysis process [64].

**Agitation rate**: It also affects the process by reducing the ethanol inhibition on cells, thereby, enhancing sugar consumption. The nutrient’s permeability to the cells and ethanol removal from the interior of the cells is increased by agitation. An RPM (revolution per minute) rate of 150 to 200 is suitable for fermentation with yeast cells [143]. Moreover, the excessive agitation rate also limits ethanol production by reducing the metabolic activities of the cells [144].

### 7.2. Nutritional parameters

**Substrate concentration**: Initial sugar concentration affects the hydrolysis process as an excess of feedstock can cause substrate inhibition, leading to the incomplete conversion of polymers to sugars, resulting in low sugar yields. High sugar concentration also affects the fermentation process and microbial activity. Many studies have observed that the high sugar concentration induces osmotic pressure in the microbial cells, reducing the efficiency of ethanol fermentation [141,145]. Increased sugar concentration and low water activity affect the growth and viability of the cells, which leads to reduced ethanol fermentation rates. Lin et al. [141] also reported that the higher initial glucose concentration (300 Kg/m^3^) at uncontrolled pH resulted in low conversion efficiency. However, the significant association between the sugar concentration and fermentation pace is more complex. The generic relation states that sugar concentration increased the fermentation rate up to a specific limit [146].

**Inoculum size**: The size of inoculum does not affect the bioethanol concentration, but affects sugar consumption and ethanol productivity. Reduced inoculum size diminishes the rate of ethanol production [144], while the increase in inoculum size over the ideal level does not increase the yield of the fermentation process [139]. Zabed et al. [137] mentioned that a rise in the cell number from 10^4^ to 10^7^ cells/ml enhanced ethanol production, but no remarkable difference was observed on rising in cells from 10^7^ to 10^8^cells/ml.

**Nutritional components**: Nutrients plays a significant part in the production as microorganism involved in the fermentation process also has some nutritional requirements to grow. The essential nutrients includes carbon and nitrogen sources, however, some micronutrients like zinc, magnesium, manganese, etc., are also required for yeast growth and ethanol fermentation. Many physiological and metabolic activities, growth, and yeast enzymes have been related to magnesium and positively affect ethanol efficiency [147]. Xu et al. [148] studied the effect of eight different metal ions (Na+, Mg^2^+, K+, Ca^2+^, Mn^2+^, Fe^2+^, Cu^2+^, Zn^2+^) on yeast fermentation and found that Mg^2+^, K+, Ca^2+^ affcted positively while, Fe^2+^, Cu^2+^, Zn^2+^ exhibited remain neutral and no sign of any adverse effect was reported. A high dose of manganese imposed toxic effect on yeast and decreased fermentation efficiency. At a high dose of Cu^2+^, Zn^2+^, the growth of *S. cerevisiae* was completely suppressed. Ghazanfar et al. [149] optimized the physical and nutritional parameters by one factorial at a time (OFAT) and central composite design (CCD), respectively. The study reported that 0.25 g/L yeast extract, 0.25 g/L (NH4)_2_SO_4_, 0.1 g/L K_2_HPO_4_, 0.09 g/L MgSO_4_, 8% substrate, 40 IU/g commercial cellulase, 1% *S.cerevisiae* inoculum, and pH 5 are optimum for maximum ethanol production (72 g/l) from alkali pretreated *Bombax ceiba*.

**Reducing agent**: The capacity of a solution to withstand oxidation-reduction processes can be defined by reducing agent. They aid in regaining the NADH from NAD+ because it is used in the production of alcohol from aldehydes. Babu [150] observed that dithiothreitol (reducing agent) addition can increase ethanol concentration with media supplemented with 0.1% yeast extract.

## 8. Ethanologenic microorganisms

Many fungal and bacterial strains are available that can ferment sugars and produce ethanol. Each microbial strain has different choices to ferment hexose or pentose sugar. The microorganism selected for the fermentation process in ethanol production should possess some traits that make it more suitable. They should have, a) high ethanol tolerance, b) utilized both C6 and C5 efficiently, c) provided high ethanol yield and productivity, d) stenothermal and survive at a broad pH range, e) osmotolerant, and f) tolerant to inhibitors [125]. Researchers are working on finding the wild strain with these traits and using different techniques to modify the microbe for better ethanol production. The following are examples of some wild strains considered in the process to ethanol production.

***S. cerevisiae***: Several thousand years ago, the brewery and wine industries used the strain for alcohol production, but today, it is widely used to produce fuel ethanol from different bioresources. *S. cerevisiae* appears as a good option for fermentation in ethanol production with its wide range of pH tolerance that makes the process less susceptible to infection [143]. Being a mesophilic strain, it can survive at an optimum temperature range of 22- 29ºC, however, it fails to grow at a temperature higher than 35ºC. Moreover, a broad range of C6 sugars, i.e. glucose, galactose, fructose, maltose, etc. can be fermented with the strain. It also possesses high growth rate, proficient glucose utilization, high ethanol production, and tolerance to high ethanol concentration and other inhibitors [151]. The glycolytic pathway is utilised for conversion of glucose, which produces two moles of pyruvate on utilization of one mole of glucose mole. Further reduction in the process gives two moles of ethanol from the generated pyruvate (2 moles) [64]. Two ATP molecules are also generated during the Embden-Meyerhof pathway (EM). However, its inability to utilize pentose sugars and incapacity to retain at higher temperatures and higher concentrations of inhibitory compounds are the key hurdles towards its utilization for bioethanol production [152].

***Z. mobilis***: A gram-negative bacterium survives at 30-39 ºC with neutral pH. The baterial strain can ferment starch and sugar hydrolyzate and produce ethanol efficiently. In contrast to *S. cerevisiae*, it has high glucose uptake and high ethanol yield and productivity [153]. It metabolizes glucose by the Entner-Doudoroff pathway (ED) to produce pyruvate converted to ethanol and CO_2_. ED pathway produces half ATP molecules as compared to the EM pathway, responsible for low biomass yield [154]. The disadvantage of *Z. mobilis* is its incompetence in utilizing both sugars, i.e. C6 and C5, resulting from cellulose and hemicellulose hydrolysis [155].

***Kluyveromyces marxianus***: The thermotolerant specie, is able to thrive at temperatures higher than 40ºC and can produce ethanol using hexose and pentose sugars. It has a rapid growth pace and is tolerant to the high concentration of inhibitory compounds. Since it could thrive at higher temperatures, which make it suitable for SSF and CBP fermentation. Moreover, the capability to utilise C6 and C5 sugars, made it ideally suited for ethanol production from lignocellulosic biomass [156].

***Escherichia coli***: The gram-negative anaerobic bacterium has the advantage of efficiently consuming hexose and pentose sugars. Moreover, its capability to tolerance higher level of toxic substances makes it more ideal for bioethanol production. Its mechanism is widely studied and shows the phenomenon of CCR (carbon catabolite repression) that regulates the microorganism to use the preferred carbon source. Due to the CCR mechanism, sugars are consumed sequentially, which limit the overall ethanol yield. The strains are genetically modified for the co-fermentation of the sugar mixture to enhance the yield and repress the regulatory mechanism of CCR [157].

***Klebsiella oxytoca***: The ethanol-producing organism can grow at a temperature of up to 35ºC and pH of as low as 5. It also can directly use cellobiose and cellotriose along with C5 and C6 sugars, thereby eliminating the use of β-glucosidase and xylosidase. Compared to *Z. mobilis*, and *E. coli*, *K. oxytoca* has a more comprehensive range of substrate utility [158].

***Clostridium species***: Many species of *Clostridium* is present such as *C. thermocellum, C. stercorarium, and C. raminisolvens*, etc., that utilize cellulose, hemicellulose, and starch. Thermophile *C. thermocellum* shows an incredible growth rate and consumes crystalline cellulose with the help of a multi-enzyme complex called ‘cellulosome’, however, it does not utilize pentose sugar [159]. Mesophile species like *C. cellulovorans and C. phytofermentans* utilize both cellulose and xylan. *C. phytofermentans* can grow at 35–37 ºC and efficiently consume starch, xylan, cellulose, pectin, cellobiose, arabinose, fructose, glucose, and galactose, and produce products like ethanol, acetate, formate and lactate [160].

***Pichia stipis***: The pentose fermenting microbe that carries out fermentation at an optimum temperature of 25–33ºC and pH range of 4.5–5.5. The strain can also ferment hexose sugars and owns both high, and low-affinity symport systems worked according to the concentration of sugars. When glucose is present in high amounts, it utilizes more glucose than xylose due to noncompetitive inhibition caused by a low-affinity symport system. Pentose utilization is more when the glucose level is low because of the high-affinity symport system of the strain [161].

***Candida tropicalis***: *C. tropicalis* is a significantly beneficial microbe for the ethanol generation for commercial purposes as it can directly ferment starch at a slow pace [162]. *C. tropicalis* can ferment the starch into ethanol due to glucoamylase enzyme production, thus eliminating the separate saccharification step [163]. This makes *C. tropicalis* more suitable for production of ethanol at industrial scale. More studies regarding ethanol production using different microbes from variable feedstocks are given in Table 4.

**Table 4.** Different microbes used in ethanol fermentation

Microbial strain	Feedstock	Yield	References
*E. coli* MM160	*Agave lechuguilla*	73.3%	Diaz-Blanco et al.[164]
*E. coli* MS04	Corncob	80.%	Pedraza et al.[165]
*E. coli* FBR5	Corn stover	92.2%	Saha et al.[166]
*K.marxianus*CBS1555	Jerusalem artichoke tuber	70.2 g/L	Kim et al.[167]
*K. marxianus*CECT10875	Barley straw	70%	García-Aparicio et al.[168]
*Z.mobilis* CHZ2501	Cassava	93.5%	Choi et al.[169]
*Z.mobilis* ATCC 29,191	Sweet potato	92.4%	Zhang & Feng [170]
*S.cerevisiae* MTCC 174	Rice straw	0.50 g/g	Singh &Bishnoi [171]
*S.cerevisiae* ATCC 24,859	Potato waste	92.08%	Izmirlioglu&Demirci [172]
*C.tropicalis* ATCC 13,803	Rice straw	0.2 g/g	Oberoi et al.[173]
*C.tropicalis* NBRC 0618	Olive prunings	0.44 g/g	Martín et al. [174]
*P.stipitis* DSM 3651	Sugarcane bagasse	0.3 g/g	Canilha et al. [175]
*P.stipitis* DSM 3651	Wheat straw	0.45 g/g	Bellido et al.[176]

## 9. Technologies to improve bioethanol production

### 9.1. Genetic engineering

This method is used to modify the biomaterial structure or the microorganism involved by inserting or knocking out the gene of interest to enhance the process output. This technique can provide multiple benefits like removing the pretreatment step, increasing sugar content, reducing the cost of cellulase enzyme, and providing co-utilization of sugars through engineered microorganisms [177]. Lignin hinders the accessibility of cellulose and hemicellulose for bioconversion. Modification in the structure of lignin to reduce lignin content through downregulation of the enzyme required in the lignin biosynthesis route can eliminate the pretreatment step. Likewise, sugar content increase can be achieved by opting for the following approaches: diverting carbon from lignin production, modifying plant growth regulators, and delaying flowering. It has been assumed that suppressing flowering genes will increase biomass production [178]. The price of the cellulase enzyme reduces up to 5 folds if plants are modified to express the enzyme [179]. Also, expressing cellulase genes in yeast helps carry out simultaneous saccharification and fermentation (SSF) [180]. Many fermenting microbes (*S. cerevisiae* and *Z. mobilis*) do not utilize pentose as carbon, affecting overall ethanol yield. This limitation can be overcome by modifying the fermenting microbes. *E. coli* is an efficient ethanologenic microorganism, however, provides low yield due to generation of other organic compounds from sugars instead of ethanol. On modification, *E. coli* overexpressed the enzymes (alcohol dehydrogenase and pyruvate decarboxylate) that resulted in enhanced ethanol production [181,182]. Chou et al. [183] co-expressed the *ictB* and *ecaA* genes in *Synechococcus elongatus* PCC7942 and obtained 202.7 mg/L ethanol production. Zingaro et al. [184] expressed the *GroESL* gene in *E. coli* and increased its ethanol tolerance up to 6%. In order to enhance ethanol output and tolerance, genetic engineering has been performed on a variety of microorganisms, as shown in Table 5.

**Table 5.** Different genes expressed or knockout in the microorganisms to increase the ethanol tolerance and its production.

S.No.	Microorganism	Gene expressed/knockout	Results	References
1.	*S.elongatus* PCC7942	*ictB* and *ecaA* (coexpression)	202.7 mg/L ethanol production	Chou et al. [183]
2.	*E. coli*	*GroESL*	6% ethanol tolerance	Zingaro et al.[184]
3.	*S. elongatus* PCC7942	*glgC* knockout and *pdc-adh* genes expressed	3856 mg/L ethanol yield	Velmurugan&Incharoensakdi [185]
4.	*S. elongatus*PCC7942	Co-expression of *ecaA*, *ictB*, and *acsAB* genes	Increased carbohydrate productivity and reached 7.2 g/L of ethanol concentration	Chow et al.[186]
5.	*Enterobacter aerogenes* ATCC 29,007	Deleting *ldhA* gene and overexpression of *adhE*	Ethanol yield was achieved 0.48 g/g	Thapa et al.[187]
6.	*S. cerevisiae*	*Rpb7* of RNA polymerase II	96.58 ± 1.14% ethanol yield	Qiu& Jiang [188]
7.	*Synechococcus* sp. PCC7002	Two *glgA* genes were targeted and introduced two *pdcZM-slr1192* cassettes	Ethanol production was 2.2 g/L (10 days)	Wang et al.[189]
8.	*S. cerevisiae*	*Sestc* cellulase gene introduced	8.1 g/L ethanol concentration and increased about 57.86-fold	Yang et al.[190]
9.	*S. cerevisiae*	*sestc* expression and knockout of *hxk2* gene	37.7-fold increase was achieved	Yang et al.[191]
10.	*S. cerevisiae*Y294	*BGL1* gene expressed	Strain Show exponential growth on cellobiose and glucose and increased β- glucosidase activity	McBride et al.[192]
11.	*S. cerevisiae*	Co-overexpression of *tps1* and *ari1* genes	Engineered strain tolerated upto 14% ethanol	Divate et al. [193]

### 9.2. Adaptive evolution

This approach improves the microorganism’s ability to survive under stressed conditions. In this method, the microorganism is cultured under specific conditions for a long time, i.e. from weeks to years, improving the phenotype of microbial species. High ethanol concentration is toxic to yeast and affects the cellular protein and plasma membrane fluidity, and impairs the transport system. The ethanol production rate can be enhanced by increasing the tolerance of the yeast [194]. Zhang et al. [195] conducted adaptive evolution to enhance ethanol production in which *S. cerevisiae* was exposed to multiple stresses, i.e. freeze-thaw treatment, ethanol, and osmotic stress resulting in a robust strain that had high tolerance toward ethanol and osmotic pressure than the wild strain. Novelli Poisson et al. [196] used an adaptive evolution methodology to improve the activity of *Scheffersomyces stipitis* for the production of ethanol. The strain was exposed to osmotic and ethanol stresses. The results showed that the evolutionary *S. stipitis* strain could be used for ethanol production from non- detoxified hydrolyzate due to increased ethanol and osmotic tolerance. Yan et al. [197] evolved *Z. mobilis* strain with improved inhibitor (phenolic aldehydes) tolerance by 6.3 fold and enhanced ethanol fermentation by 21.6% laboratory adaptive evolution for 198 days. Different studies on adaptive evolutions of various microbes and their effect on ethanol yield are represented in Table 6.

**Table 6.** Adaptive evolution in different ethanol-producing microbes.

Microbial Species	Adaptation	Ethanol Yield	References
*Z. mobilis* ZMF3–3	Furfural and Acetic acid tolerance	94.84%	Shui et al.[198]
*E. coli* SZ470	Higher Ethanol tolerance (350 generations)	94%	Wang et al.[199]
*S. cerevisiae ISO12*	Combined tolerance for inhibitors and temperature (280 generations)	0.38 g/g	Salinas &Grauslund [200]
*Z. mobilis* AD50	Simultaneous utilization of glucose and xylose (50 transfers)	0.49 g/g	Sarkar et al.[201]
*S. cerevisiae* YF10–5	Improved osmotic pressure and ethanol stress tolerance	93.95%	Zhang et al.[195]
*S. cerevisiae*	Improved xylose fermentation	0.43 g/g	Shen et al.[202]
*E. coli* SSK42	Improved specific growth rate and ethanol yield (42 days)	89%	Jilani et al.[203]
*C. thermocellum* LL1210	Improved ethanol yield	75%	Tian et al.[204]
*S. cerevisiae* CC156	Low pH and lignocellulosic inhibitors tolerance (709 generations)	0.45 g/g	Narayanan et al.[205]

## 10. Bioethanol commercialization and scale-up

The bioethanol biorefinery concept indicates the application of biomass to produce sustainable bio-products or bioenergy along with bioethanol by using an integrated approach. First-generation bioethanol production has developed at a large scale. A major portion of total bioethanol production is contributed from United States and Brazil-based 1G ethanol plants. The lignocellulosic biomass components are fractionated and utilized for ethanol manufacturing and other valuable materials like xylitol, sorbitol, succinic acid, furfural, vanillin, etc. in second-generation bioethanol biorefinery. All these aforementioned products have market value and are commercialized on a large scale. In Europe, 43 biorefineries were based on lignocellulosic biomass [206]. The allocation of lignocellulosic biorefinery is done for a number of reasons: to endure renewable and sustainable energy; to save foreign exchange reserve by diminishing the reliance on the imported crude petrol and increase the economic growth as a whole; to get a low carbon footprint and ecologically sound environment; and for the establishment of the circular economy [207]. The Indian Oil Corporation has also announced their intension to build a cellulosic ethanol plant with a 63 million liter capacity by using a dilute acid pretreatment process. TATA has also envisaged building an ethanol plant with a capacity of 100 KL at Bargarh, Odisha, India [208].

Likewise, third-generation algal biorefinery produces ethanol, methane, biodiesel, syngas, and biohydrogen on following the process of fermentation, anaerobic digestion, transesterification, gasification, and biophotolysis, respectively. For increasing the efficiency of ethanol production, cellulose-rich waste streams are considered as a propitious technique that focuses to optimize the process stream for achieving the goal of a 6–8% annual increase in liquid biofuels by 2050 [209]. Substitutes like bio-oil and biomethane were explored in macroalgae biorefinery waste streams that unfolded exploration of novel methods in research and energy sector at commercial level. Many patents have been documented recently in biofuels from macroalgae to commercialize the process, however, work at a slow pace. Six patents were documented for the processes that resulted in various biofuels and bio-products. The aforementioned patents include renewable chemicals, biofuels, fermentation sugars, acids, and alcohols (US9688595B2); Levulinic acid, hydroxymethylfurfural and formic acid (US9452993B2), bioethanol (CN101024847, US2013005009A1); biofertilizers and agricultural feed (CN101024847, US2013005009A1, US10000579B2); industrially applicable bioproducts such as lipids, agricultural feed, pigments, hydrocolloid agar from red seaweed (US10000579B2) [210–215]. Exploration of bioenergy from macroalgae was begun in 1970 and its practical facets (pilot projects) were noticed after 2010. Some initiatives like the SeaGas project, Macro fuels, MacroBio crude, and Global seaweed have been completed. There are about 32 funded projects (2018-till now) by the Governments of the United States, United Kingdom, Europe, Australia, and New Zealand on the production of the final product with the capability to commercialize downstream byproducts [216]. In totality, it has been noticed that bioethanol derived from 1G substrate is very much commercialized in US and Brazil but 1G substrate is not suitable for large scale in developing countries like India. Therefore, to enhance production at the global level, the circular economy concept needs to be followed to produce bioethanol at a large scale from 2G and 3G feedstocks. Commercialization of bioethanol from 1G, 2G, and 3G feedstocks require process integration and exploration and incorporation of novel techniques such as nanoparticles and genetic engineering.

## 11. Life cycle analysis

The process of assessing the impact of a product throughout its life, from the extraction of raw material to its use and final disposal, is called Life Cycle Analysis (LCA). Different generations of bioethanol have different impacts on the environment depending upon the feedstocks and the methods used. In the case of GHG emissions, a neat biofuel at the time of blending has 50% lower emissions throughout the lifecycle than fossil fuels; however, the net emissions may occur at the steps of cultivation, feedstock processing, transport and conversion [217]. Using bioethanol as fuel can certainly reduce GHG emissions from the transportation sector. There was a total reduction of 0.5 billion tons of CO_2_-eq emissions due to the use of bioethanol during the period from 2008 to 2018, and it is also predicted that it can lead to a further reduction of 160 billion tons of CO_2_-eq by 2030, with the majority of bioethanol produced from 1G feedstocks [218]. However, the land-use change associated with the cultivation of first-generation feedstocks can significantly affect the emissions during the lifecycle of bioethanol. Conversion of grasslands/forestlands to croplands had a net emission of 0.002–0.009 kg CO_2_-eq/MJ for corn bioethanol [219].

Similarly, Scully et al. [220] reported an emission of 0.0039 kg CO_2_-eq/MJ due to land-use change. Direct land-use change from perennial croplands to annual cropland produces 0.02 and 0.0179 kg CO_2_-eq per MJ of bioethanol generated from cassava and sugarcane, respectively [217]. Apart from land-use change, 1G feedstocks have other impacts such as eutrophication potential, pesticides pollution and water utilization. The eutrophication potential of bioethanol is mainly due to wastewater generated during the production and fertilizers leaching into water bodies. The eutrophication potential of cassava and sugarcane was found to be 0.0058 and 0.0007 kg P-eq per GJ, as reported by Papong et al. [217]. Similarly, feedstocks like sugar beet and wheat can increase the eutrophication potential of bioethanol [221,222]. The second-generation bioethanol tends to have lower carbon emissions and lower ozone layer depletion, while having a positive or negative effect on other impact parameters such as eutrophication, acidification, human health and photochemical smog [223]. Maga et al. [224] performed LCA of first and second-generation bioethanol from sugarcane and reported that second-generation bioethanol had a reduction in each impact category. Common reed, natural wetland grass, was evaluated for bioethanol production; the LCA results showed that the greenhouse gas emission intensity was 0.15 kg CO_2-_eq/MJ and eutrophication potential was −0.0011 kg PO_4_^3+.^eq/MJ, indicating sustainability of common reed for bioethanol production [225]. Surplus rice straw available in India, when converted to bioethanol, has the potential to reduce 11,498–14,498 kt CO_2_-eq by the year 2030–2031, when renewable electricity replaces fossil fuel-driven electricity [226]. However, in a scenario where the processing energy is supplied by coal, the net emission from the rice straw bioethanol would be very high [227]. The third-generation bioethanol also appears to be a suitable biofuel. In terms of CO_2_ emissions and energy balance, microalgal bioethanol manifests itself as a favorable alternative fuel and other than that, it has low water and land footprints as compared to other energy crops [228].

Bioethanol performs better than fossil fuels in the impact category of global warming potential due to zero net carbon emissions during combustion. However, based on the process, the other impact categories may be positively or negatively affected. It is tough to compare the results of different LCA studies because of different approaches, like different functional units, allocations, and system boundaries.

## 12. Current challenges and prospects

Different feedstocks were used for ethanol production with different process steps. The first generation creates a food versus fuel debate. So, lignocellulosic biomass can be used as a plentiful renewable source. However, the recalcitrant nature of its structure poses hurdles to extracting sugars from the biomass. Various pretreatment technologies can break its structure, but the pretreatment process quenches a lot of energy. Therefore, more research is needed to decrease the energy consumption during the whole ethanol production process. Various expensive enzymes are used in biological pretreatment and enzymatic hydrolysis, so there is a need to explore and produce low-cost or thermally stable enzymes in future also. The modified plant biomass can reduce the price of pretreatment and hydrolysis [229]. To increase the ethanol production yield, the researchers have made many recombinants or genetically modified strains. Many strains like *S. cerevisiae* can ferment hexose sugars very efficiently, but the feedstock for ethanol production contains both hexose and pentose sugars. Therefore, to isolate or find the microorganism that can metabolize both the C5 and C6 sugars efficiently, is one of the potential challenge towards bioethanol production [33].

Another challenge is the existence of inhibitory substances in the hydrolyzate that can adversely impact the growth and the viability of the microbial cells and their metabolic pathway throughwhich sugars are converted to ethanol. Strains tolerant to the inhibitory substances are significant for the scale-up process, which represent the area where research investigations are required. Third-generation biofuel technology has not yet been commercialized entirely due to its considerable expenses. Therefore, there is a need to explore the financial and inexpensive conversion technologies. Fourth-generation biofuels are in the growing stage and facing challenges like lack of information about the genes and biology of the microbial species. Future studies are needed to explore the best suited algal species. Moreover, research investigations are required to evaluate the as-yet-unknown effects of using GMOs as feedstock and also to explore most sustainable and feasible technology for the effective and efficient use of bioresources for bioethanol production.

## 13. Conclusions

The growing energy demand will lead to a move toward renewable energy resources. Different countries have made their biofuel policies, considering the rapid depletion of energy resources. Each generation of biofuel production consists of different process steps according to the biomass structure and composition. Despite the abundant feedstock for bioethanol production, some technological barriers can still affect its production efficiency. Therefore, advanced technologies like genetic modification and adaptive evolution have been used to enhance the yield and overcome the barriers. The studies conducted at various levels have advocated the suitability of these tools to boost the production of biofuels. However, the overall production cost is high, which needs to be lower down for its commercialization and large scale prodcution. Technical advancement is essential to cut costs by reducing processing steps. Moreover, genetically modified organisms (GMO) are required to be developed that could efficiently metabolize both sugars (hexose and pentose) present in the feedstocks to enhance yield and production of bioethanol.
